# Fatal Vaccinia during Whooping Cough

**Published:** 1846-02

**Authors:** 


*Fatal Vaccinia during Whooping Cough.*—Dr. Vandervoort reported a fatal case of vaccinia during whooping cough. The child was apparently of a healthy constitution, and vaccinated with the view of cutting short the disease. He was called to see the child on the third or fourth day, when hæ-

morrhage had occurred at the point of vaccination. The hæmorrhage had much increased during the paroxysm of coughing. This state of things continued for three weeks, notwithstanding the treatment, which had consisted in the application of cold and astringents, the child dying at length in a state of perfect anemia.—*Ibid.*

